# From Ideal to Real: Attachment Orientations Guide Preference for an Autonomous Leadership Style

**DOI:** 10.3389/fpsyg.2022.728343

**Published:** 2022-02-21

**Authors:** Dritjon Gruda, Konstantinos Kafetsios

**Affiliations:** ^1^School of Business, Maynooth University, Maynooth, Ireland; ^2^Palacky University, Olomouc, Czechia; ^3^School of Fine Arts, Aristotle University, Thessaloniki, Greece

**Keywords:** leadership, attachment theory, personality, indivdual characteristics, implicit leadership theories

## Abstract

Autonomy is a key characteristic of attachment relations that varies as a function of attachment orientations and is also a key personality characteristic of leadership perceptions. In the presented research, we reasoned that the relationship between attachment and autonomy-related preference for specific leaders and leadership behavior would be a function of individuals’ insecure attachment strategies. We tested our hypotheses in two studies. Study 1 used Multiple Indicators Multiple Causes (MIMIC) modeling to test expectations based on a cross-sectional design, while Study 2 utilized a vignette-based experimental design. We find that anxious individuals attributed less positive evaluations to an autonomous leadership style (Study 1), while avoidant persons attributed higher leader competence to an autonomous leader description (Study 2). Compared to less anxious participants, highly anxious participants attributed lower competence to the autonomous leader description. By examining how individual differences in attachment orientations can indirectly influence the ideal leader categorization process, the present set of studies lends support to the importance of attachment orientations and related working models in leader perception and contribute to the literature on leader-follower fit. Using a survey and experimental approach, we examine how followers’ attachment schemas can shape the leader influence process, specifically concerning a preference for an autonomous leadership style.

## Introduction

Individuals form mental representations of desired leadership attributes and behavior they would like to see in their leaders which form the foundation of implicit leadership theories (ILTs; Cronshaw and Lord, 1987). Largely unconsciously held, ILTs allow individuals to distinguish leaders (those that fit perceivers’ ILTs) from non-leaders (those that do not fit perceivers’ ILTs). Indeed, one of ILTs’ key functions is the easing of cognitive load by automatically and effortlessly matching perceived leader traits to the already held ideal ILTs, which, in turn, shape both preferred and non-preferred leader attributions (Schyns and Schilling, 2010). Yet, less is known about individual-level variation in followers’ leader preferences and consequent leader-follower fit (Junker and van Dick, 2014).

The present set of studies examined how individual differences in followers’ attachment orientations, varying on the avoidance and anxiety dimensions, indirectly influence preferred leader characteristics. In particular, we focus on a preference for and attributions, such as perceived competence, to leaders who exhibit an autonomous or a less autonomous leadership style. Autonomous leadership constitutes one of several attributes of an ideal leader prototype (House et al., 2004) and adult attachment orientations are particularly suited to account for followers’ idealized and preferred leadership mental images (Gruda and Kafetsios, 2020). We propose that perceptions of leaders who exhibit higher autonomy characteristics (e.g., independence) are associated with followers’ avoidant and anxious attachment orientations in theoretically meaningful ways. In doing so, the present set of studies add to the understanding of socio-cognitive processes relatied to followers’ attachment orientations (Harms, 2011) and, consequently, leader-follower fit (Epitropaki et al., 2013).

### Preferred Leaders and Attachment Orientations

Whenever followers engage in leader identification decisions, they implicitly compare perceived leaders to the mental image of an ideal leader (Shondrick et al., 2010; Foti et al., 2012). A good match between the perceived leader and followers’ cognitively represented ideal image of how a leader should be and should not be like will result in more favorable “reactions toward [and interactions with] the leader” (Foti et al., 2012: 1). There are different types of leader schema, including ideal, typical, effective, political (Epitropaki et al., 2013). As noted by Junker and van Dick (2014, p. 3) “ideal leader prototypes differ substantially from typical [leader] prototypes” in that “ideal prototypes can be quite extreme and be more on the periphery of the [leader] category”. Put differently, ideal leadership behavior does not equate to actual (or typical) leader behavior; rather, ideal leadership is based on followers’ desired expectations of how leaders should or should not be like. However, this is not to say that ideal leader prototypes are somehow less important than typical leader prototypes in leader-follower interactions.

An ideal leader schema does not only include characteristics associated with an ideal leader, but also characteristics that an ideal leader should not possess (i.e., characteristics of non-leaders). In support of this distinction is evidence that effective leadership does not equate to favorable leadership, in that leader attributes can be categorized both into effective and unfavorable or vice versa (Schyns et al., 2011). Therefore, attributes that describe typical leaders do not necessarily describe, or might even be irrelevant to, ideal leaders (Junker and van Dick, 2014). Hence, ideal leader prototypes and the respective ideal leader categorization process are vital in most leader-follower interactions. The studies presented in this paper specifically focus on the ideal leader prototype and preferred leadership behaviors.^1^

Ideal leader schemas are closely associated with self-schemas. Previous research has shown that ideal leader prototypes are established in a self-serving manner (e.g., Dunning and Hayes, 1996). Specifically, individuals are likely to include characteristics that are similar to themselves in their ideal leader prototype. That is, individuals are likely to merge their self-concept with the leader schema to produce an ideal leader prototype. For example, outgoing and social individuals are more likely to look for sociability in their ideal leaders than individuals who do not consider themselves outgoing. Likewise, concerning negative characteristics, followers are likely to de-emphasize their own negative characteristics in the definition of their ideal leaders (Foti et al., 2012). It is this self-perception factor in the ideal leader categorization process that provides a well-founded link to attachment theory and adult attachment orientations.

Attachment working models involve self and other schemas, and the aggregation of memories of significant others across relationships, that lead to forming impressions and expectations of others in particular roles such as leadership roles (Kafetsios and Gruda, 2018; Gruda and Kafetsios, 2020). An avoidant working model involves a negative view of and distance from others, with an emphasis and focus on the self, while an anxious attachment working model involves a more positive view of others compared to the self. This positive view of others typically leads anxiously attached individuals to experience a strong need for intimacy and increased proximity and closeness to important others (Mikulincer and Shaver, 2007).

Several studies have demonstrated that followers’ attachment orientations are associated with followers’ leadership perceptions and leader prototypes. For example, Keller (2003) proposed that follower attachment orientations influence idealized leadership mental images, as these images mirror descriptions of parental traits. Similarly, Hansbrough (2012), found that anxious followers are biased in evaluating their leaders as more transformational, in line with their dominant attachment orientation. More recently, Gruda and Kafetsios (2020) have shown that attachment orientations predict the transfer of leadership expectations. These studies highlight the nature of leader-follower perceptions and leadership categorization processes (Junker and van Dick, 2014). In the present paper, we focused on a particular characteristic of idealized leadership, namely, autonomous leadership.

### Attachment Orientations and Autonomous Leadership

Autonomy is a seminal aspect of relationships and is closely linked to attachment orientations and the corresponding working models of self and others. Attachment and relations theorists stress the significance of autonomy in child-caregiver interactions. Adolescents and children with secure attachment orientations are involved in interactions with parents characterized by healthy autonomy as reflected in cognitive and emotional indicators of those interactions (Allen et al., 2002; McElhaney et al., 2009). And while avoidant adolescents exhibit low levels of relatedness in interactions with their parents, anxious attachment is associated with over-engaging parenting (Allen and Hauser, 1996). The anxious–ambivalent parenting style also is characterized as ‘intrusive parenting’ (Rholes et al., 1995). These interactions with an authority figure shape expectations (working models) of autonomous leadership and interactions with leaders in later life, depending on which attachment orientation is the most dominant.

Regarding ideal leadership, autonomy is a “leadership dimension that refers to independent and individualistic leadership attributes” (House et al., 2004, p 14). An autonomous leader is characterized by “a high degree of independence from superiors and a high degree of social distance from subordinates, a tendency to be aloof and to work alone” (House et al., 2004, p. 7). An autonomous leader may also display self-governing behavior, for example, acting separately from others (Dorfman et al., 2012, p. 508). Autonomy also constitutes a key leadership characteristic to do with self-determination (Chiniara and Bentein, 2016).

### Avoidant Attachment Working Models

Avoidant attachment is characterized by an emphasis on autonomy and self-reliance, a reluctance to trust others, and a relatively low tolerance for interpersonal intimacy and interdependence (Popper and Amit, 2009). Others are also often devalued to inflate avoidant individuals’ own capabilities and self-worth (Rom and Mikulincer, 2003). As Richards and Hackett (2012, p. 689) point out: “followers high in attachment avoidance will behave in ways aimed at verifying their self-concept of a socially distant ‘lone wolf.” Hence, we hypothesize that characteristics such as self-reliance and autonomy are part of avoidant individuals’ ideal leader prototype, resulting in a higher preference for such a leadership style:

*H1*: Higher avoidant attachment orientation is associated with a higher preference for and higher positive attributions to an autonomous leadership style.

### Anxious Attachment Working Models

Anxiously attached individuals prefer both physical and emotional closeness and with others and begin to resort to increased proximity and support-seeking behavior, to overcome their constant worry over abandonment and fear of loneliness (Popper and Amit, 2009). Anxious individuals also increasingly look to others for help and safe-haven, oftentimes inflating others’ abilities and capabilities in line with a positive model of others (Bartholomew and Horowitz, 1991). Hence, in terms of ideal leadership, anxious individuals likely envision and are drawn to leaders who strengthen them as followers and who constitute a possible safe-haven (Rom and Mikulincer, 2003). Hence, we argue that characteristics such as self-reliance and autonomy do not form part of anxiously individuals’ ideal leader prototype, resulting in a lower preference for such a leadership style:

*H2*: Higher anxious attachment orientation is associated with a lower preference for and less positive attributions to an autonomous leadership style.

## Overview of Studies

Study 1 provided a first examination of the preference for and positive attributions to an autonomous leadership style using the ideal leader prototype attribute list by House et al. (2004). Study 2 experimentally tested whether attachment orientations shape competence attributions to an autonomous or less autonomous leadership style thus revealing a preference for such a leadership style. In both studies we adopted a two-dimensional approach to measuring followers’ attachment orientations (Fraley et al., 2015), including the interaction between avoidance and anxiety (see Gruda and Kafetsios, 2020; Gruda and Kafetsios, 2021).

## Study 1

### Sample and Procedure

The first study followed a two-wave design, with 2 weeks between each wave. We collected a sample of 298 U.S. participant responses on Amazon’s Mechanical Turk (MTurk) platform *via* the CloudResearch dashboard. To lower the likelihood of MTurk workers to click through surveys randomly, we included: (a) various pre-tested attention check questions including both direct and bogus items according to best practices (DeSimone et al., 2015), (b) time screens to prevent participants from answering items too quickly (Wood et al., 2017), and (c) included only participants who had a high approval on the MTurk platform.

In the first wave, we collected responses on individual differences, including attachment orientations, personality, and demographic questions. Most participants indicated whether their most recent job position was that of an employee (59.7%) or middle management (15.44%). Female participants accounted for 54.7% of the sample (134 males; 1 gender unknown). The average age of participants was 42.59 years (SD = 11.99), with an average work experience of 17.69 years (SD = 11.98).

In the second wave, participants were shown and indicated their agreement to the complete ideal leader prototype attribute list (House et al., 2004), to allow participants to social-cognitively contrast items against each other (Stapel and Suls, 2007) and to ensure that participants’ leader prototype is activated. All items are shown in random order to each participant, in line with House et al. (2004).

### Measures

#### Autonomous Leader Ideal Prototype

The ideal leader prototype scale instructs the participant to rate several “… behaviors and characteristics that can be used to describe leaders […] accompanied by a short definition to clarify its meaning.” Items were rated using a scale ranging from: (p. 1) “Greatly inhibits a person from being an outstanding leader” to (p. 7) “Contributes greatly to a person being an outstanding leader.”

The latent factor “autonomy” was formed comprising four behavioral trait items describing an autonomous leadership style: “autonomous” (description: acts independently, does not rely on others), “independent” (does not rely on others; self-governing), “individualistic” (behaves in a different manner than peers), “unique” (an unusual person; has characteristics of behaviors that are different from most others). The Cronbach alpha for autonomy (*α* = 0.60) equals the reliability factor in the original GLOBE study (*α* = 0.59; House et al., 2004).

#### Attachment Orientations

Attachment orientations were assessed using the adapted Experiences in Close Relationships scale (ECR) by Richards and Schat (2011). The adapted ECR scale replaced references to romantic partners in the items with “other people” or “others” and is used in more general contexts, such as organizational settings. The ECR consists of 36 items on two 18-item subscales measuring anxious (*α* = 0.96, *M* = 2.98, SD = 1.36) and avoidant attachment (*α* = 0.96; *M* = 3.88, SD = 1.4). Participants rated their agreement for each subscale on a 7-point scale ranging from 1 (strongly disagree) to 7 (strongly agree).

#### Big Five Personality

Given some overlap between attachment and Big Five personality dimensions (Noftle and Shaver, 2006), we account for these traits as well using the Mini IPIP scale (Goldberg et al., 2006). Openness to experience (*α* = 0.81), conscientiousness (*α* = 0.81), extraversion (*α* = 0.87), agreeableness (*α* = 0.85) and neuroticism (*α* = 0.81) were measured with ten items each, rated on a 5-point scale ranging from (1) “very inaccurate” to (5) “very accurate.”

*Demographics* included age, gender, and job position.

## Results

An overview of descriptive statistics and pairwise correlations are reported in Table 1.

**Table 1:** Overview and correlations of studied variables (Study 1).

		*M*	*SD*	1	2	3	4	5	6	7	8	9	10
1	Autonomy (aggregated score)	4.40	1.07	(0.60)									
*Attachment*													
2	Anxious	2.96	1.36	−0.12^*^	(0.96)								
3	Avoidance	3.87	1.41	−0.00	0.34^***^	(0.96)							
*Big 5 Personality*													
4	Openness	4.05	0.87	−0.03	−0.02	−0.18^**^	(0.81)						
5	Conscientiousness	3.76	0.91	0.02	−0.40^***^	−0.18^***^	−0.06	(0.81)					
6	Extraversion	2.54	1.1	0.03	−0.27^***^	−0.56^***^	0.31^***^	0.07	(0.87)				
7	Agreeableness	3.84	0.87	0.00	−0.12^*^	−0.60^***^	0.33^***^	0.08	0.35^***^	(0.85)			
8	Neuroticism	2.50	1.01	−0.03	0.66^***^	0.38^***^	−0.03	−0.41^***^	−0.32^***^	−0.16^**^	(0.81)		
*Controls*													
9	Age	42.59	12.00	0.06	−0.28^***^	−0.05	−0.09	0.18^**^	−0.02	0.08	−0.20^***^	–	
10	Gender	1.45	0.50	−0.05	−0.02	0.15^*^	0.02	0.01	−0.09	−0.28^***^	−0.08	−0.08	–
11	Job Position	2.13	1.57	0.06	−0.11	−0.10	0.18^**^	0.03	0.19^**^	0.12^*^	−0.12^*^	0.09	0.04

*Gender (female = 1, male = 2)*.

* *p < 0.05*;

** *p < 0.01*;

*** *p < 0.001; n = 298; reliability alphas in parentheses, where appropriate*.

### Method of Statistical Analysis

Keeping with previous studies using the GLOBE scale, we treated autonomy as a latent variable and computed a one-factor model with all autonomous leadership items loading onto a single latent factor (see House et al., 2004). The chi-square (*χ*^2^) statistic, the comparative fit index (CFI), the non-normed fit index (NNFI), and the root-mean-square error of approximation were used to assess model fit (RMSE; Bentler and Bonett, 1980, Bentler, 1990). A one-factor model without predictors was fitted, adding a direct path between the variables unique and individualistic, as they are conceptually close. This model provided the best fit (*χ*^2^ = 0.95, *p* > 0.33; *CFI* = 0.99; *SRMR* = 0.01; *NNFI* = 0.99; *RMSEA* = 0.00).

**Figure 1:**
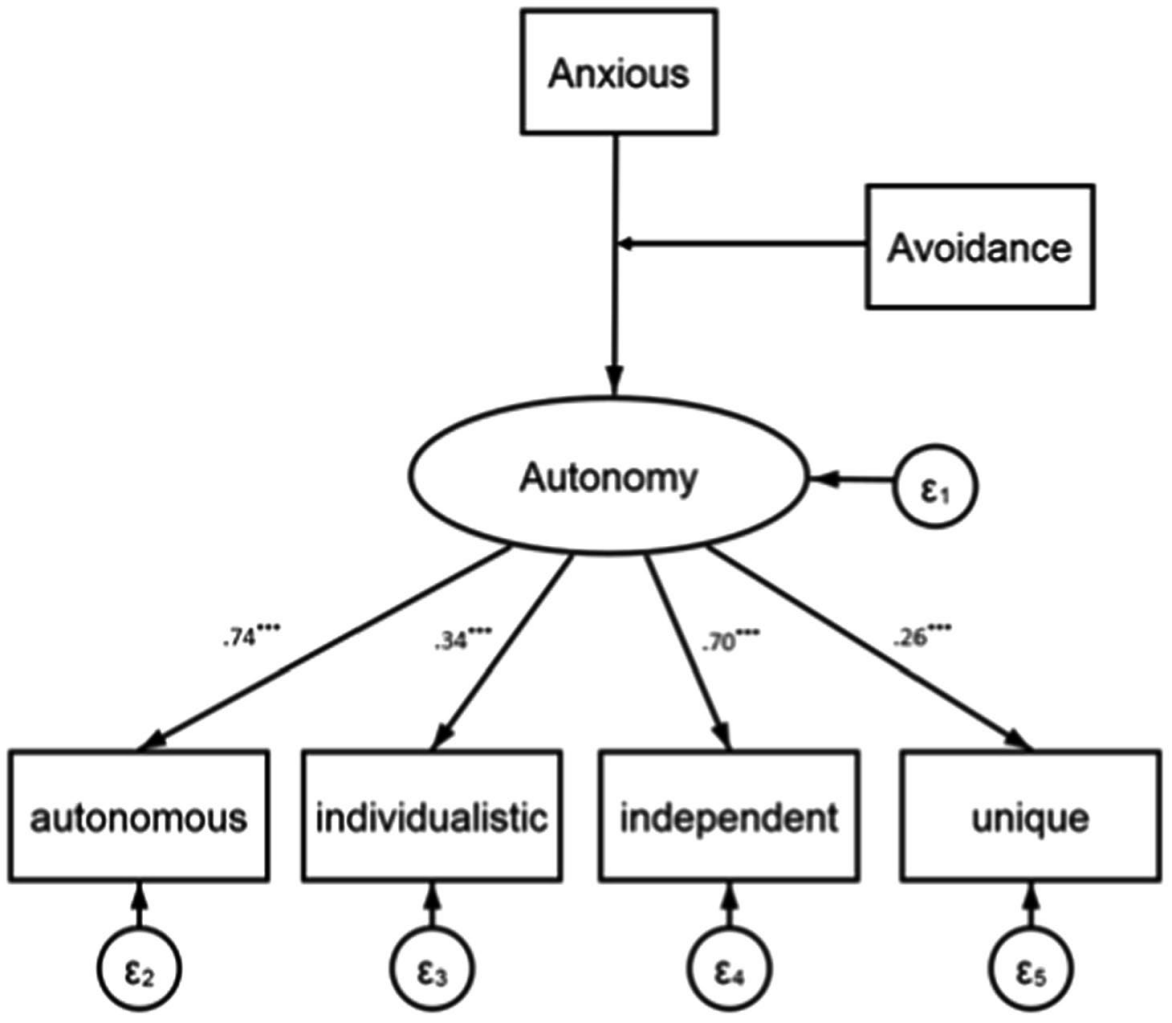
Maximum-likelihood parameter estimates for the hypothesized model without controls (Study 1). ^*^p < 0.05, ^**^p < 0.01, ^***^p < 0.001; *n* = 297; standardized coefficients. RMSEA: 0.043, CFI: 0.956, SRMR: 0.04, *χ*^2^ (11) = 17.14, *p* > 0.14.

We specified a Multiple Indicators Multiple Causes (MIMIC) model (Jöreskog and Goldberger, 1975; Muthén, 1989) due to two main reasons. Firstly, MIMIC models allow the testing of direct and indirect effects of attachment orientations on autonomy as a latent variable and the dimension’s individual items (i.e., unique, individualized, independent, and autonomous), respectively. Secondly, the MIMIC approach allows us to assess the impact of interactions using continuous predictors, such as anxious and avoidant attachment, on a latent variable within a structural equation model (Woods et al., 2009). A decent fit for the structure to the data was found with regard to autonomy and predictors (*χ*^2^ = 17.14, *p* > 0.10; *CFI* = 0.96; *SRMR* = 0.04; *NNFI* = 0.93; *RMSEA* = 0.04, see Figure 1).

A significant negative relationship between anxious attachment and preference for an autonomous leadership style was found (*b* = −0.18, *p* = 0.01). There were also indirect effects of anxious attachment on the various items of the autonomy dimension (independent: *b* = −0.15; individualistic: *b* = −0.07; autonomous: *b* = −0.18; unique: *b* = −0.04; all *p* < 0.05). On the other hand, the relationship between avoidant attachment and autonomous leadership was not found to be significant (*b* = 0.02, *p* > 0.10).

Introducing additional controls such as the Big-5 personality dimensions and demographics improved model fit (*χ*^2^ = 37.19, *RSMEA* = 0.02, *SRMR* = 0.03, *CFI* = 0.98, *NNFI* = 0.98; *p* = 0.37). To better understand these findings, we further examined our hypotheses regarding autonomous leadership using an experimental design in Study 2.

## Study 2

Study 2 expanded on findings from Study 1 testing the effects of autonomous and less autonomous ILTs in an experimental design. To assess preference for and positive attributions to an autonomous leadership style based on attachment orientations, we used perceived leader competence as a possible positive attribute. In the vignette-based experimental setting, we examined whether participants’ leader preference and degree of (higher or lower) positive attributions to autonomous leadership vary when evaluating descriptions of a highly autonomous or less autonomous manager.

### Sample and Procedure

We recruited 400 U.S. participants *via* Amazon’s Mechanical Turk to take part in this experiment. Our final sample included 193 female participants (206 males; 1 unanswered), ranging from 20–75 years of age (*M* = 36.76, SD = 10.80), with an average work experience of 15.59 years.

Participants were provided with an informed consent form and told that they would be asked to complete measures on individual characteristics. These measures consisted of the same measures as in Study 1, namely attachment (Richards and Schat, 2011) and Big Five personality traits (Goldberg et al., 2006). Secondly, participants were randomly assigned to either an autonomous leader condition or a control condition and then asked to evaluate the presented leader description on competence. Finally, a manipulation check was conducted, and participants completed demographic measures. After completion, participants were fully debriefed and thanked for their help.

#### Vignettes

Our goal was primarily to create two vignettes, identical in sentence structure and word count, examining the variance due to individual attachment orientations differences. Both vignettes counted 147 words and 8 sentences. In both vignettes we used the same introductory section to describe the leader’s back story:

*“Mark Smith is Director of Sales for a major appliance firm. Mark assumed his position two years ago following his attainment of an MBA degree with a specialization in marketing. In this position, he has gained the respect of both his subordinates and his superiors. His superiors evaluate him as a capable worker, and his subordinates have indicated that they enjoy working for him. Mark is currently in charge of 12 subordinates.”*

The section above was followed by a different story for each leader style condition. For example, the autonomous leader condition included sentences such as “*Mark achieves what he sets out and does not rely on others’ help*,” while the less autonomous leader condition includes sentences such as “*Mark achieves what he sets out and relies strongly on other’s input*.”

### Pre-test of Vignettes

To ensure that participants perceived each vignette correctly, they were asked to rate each vignette on the four items of autonomous leadership as outlined in Study 1. A subsequent t-test using the aggregated autonomy score showed a significant difference (*t* = −20.88, *p* < 0.00) between the autonomous (*M* = 4.12, SD = 0.79) and less autonomous leadership condition (*M* = 2.38, SD = 0.87). These results suggest that the manipulations had their intended effects.

### Measures

We included the same independent variable measures as in Study 1, namely attachment and Big Five personality traits. We also included age and gender as controls. Perceived leader competence was measured using one item, with answer choices ranging from (1) “not at all” to (5) “extremely.”

## Results

Correlations and reliability alphas are reported in Table 2. Using ordinary least squares (OLS) regressions with a heteroscedastic-robust estimate of the variance, we regressed the continuous variable – reflecting the degree of competence attributed to the described leader – on the manipulated variable leader condition, as well as anxious and avoidant attachment and their interaction (Table 3).

**Table 2:** Correlations of studied variables (Study 2).

		*M*	*SD*	1	2	3	4	5	6	7	8	9	10	11
1	Autonomous Leader Condition	0.50	0.50											
2	Perceived Leader Competence	4.25	0.82	−0.12^*^										
*Attachment*														
3	Anxious	3.03	1.38	−0.01	−0.10^*^	(0.96)								
4	Avoidance	3.80	1.39	−0.04	−0.02	0.51^***^	(0.96)							
5	Anxious^*^Avoidance	12.48	8.45	0.01	0.15^***^	0.89^***^	0.78^***^	–						
*Big 5 Personality*														
6	Openness	3.91	0.86	−0.03	0.13^**^	−0.22^***^	−0.20^***^	−0.22^***^	(0.78)					
7	Conscientiousness	3.69	0.94	−0.06	0.06	−0.51^***^	−0.31^***^	−0.48^***^	0.15^***^	(0.83)				
8	Extraversion	2.62	1.08	0.03	−0.11^*^	−0.33^***^	−0.55^***^	−0.47^***^	0.21^***^	0.12^*^	(0.88)			
9	Agreeableness	3.76	0.91	0.02	0.16^***^	−0.18^***^	−0.49^***^	−0.31^***^	0.37^***^	0.21^***^	0.26^***^	(0.85)		
10	Neuroticism	2.44	1.01	−0.04	−0.04	0.70^***^	0.43^***^	−0.67^***^	−0.26^***^	−0.45^***^	−0.39^***^	−0.14^***^	(0.83)	
*Controls*														
11	Age	36.76	10.80	0.04	0.05	−0.25^***^	−0.09	−0.19^***^	0.03	0.19^***^	0.01	0.19^***^	−0.17^***^	
12	Gender	1.51	0.51	−0.03	−0.03	−0.06	0.01	−0.05	0.06	−0.05	0.05	−0.23^***^	−0.18^***^	−0.09

*Gender coded as 1 (female) and 2 (male); Autonomous Leader Condition (Low Autonomous leader = 0, High Autonomous leader = 1)*.

* *p < 0.05*;

** *p < 0.01*;

*** *p < 0.001; n = 400; reliability alphas in parentheses, where appropriate*.

**Table 3:** Regression estimates (Study 2): perceived leader competence.

Variables	(1)	(2)	(3)	(4)
Autonomous Leader Condition	−0.19^*^(−2.36)	−0.21^*^(−2.54)	−0.64(−1.43)	−0.84^*^(−2.03)
Anxious Attachment		−0.39^***^(−4.09)	−0.40^***^(−3.71)	−0.37^***^(−3.46)
Avoidant Attachment		−0.18^**^(−2.67)	−0.28^***^(−3.88)	−0.27^***^(−3.50)
Anxious^*^Avoidant Attachment		0.08^***^(3.62)	0.09^***^(3.75)	0.08^***^(3.47)
Condition^*^Anxious Attachment			−0.02(−0.13)	0.01(0.04)
Condition^*^Avoidant Attachment			0.21(1.55)	0.27^*^(2.30)
Condition^*^Fearful Attachment			−0.02(−0.53)	−0.03(−0.87)
Openness	0.08(1.69)			0.06(1.18)
Conscientiousness	−0.00(−0.09)			−0.04(−0.88)
Extraversion	−0.15^***^(−3.52)			−0.15^**^(−3.01)
Agreeableness	0.16^**^(3.11)			0.17^***^(3.33)
Neuroticism	−0.06(−1.27)			−0.01(−0.30)
Constant	3.97^***^(11.28)	5.26(21.58)	5.50(22.33)	5.19^***^(10.98)
*F*	3.5	5.14	5.16	0.4.91
*R^2^*	0.08^**^	0.05^***^	0.07^***^	0.13^***^

* *p < 0.05*;

** *p < 0.01*;

*** *p < 0.001; n = 400; robust t-statistics in parentheses; unstandardized coefficients*.

**Figure 2:**
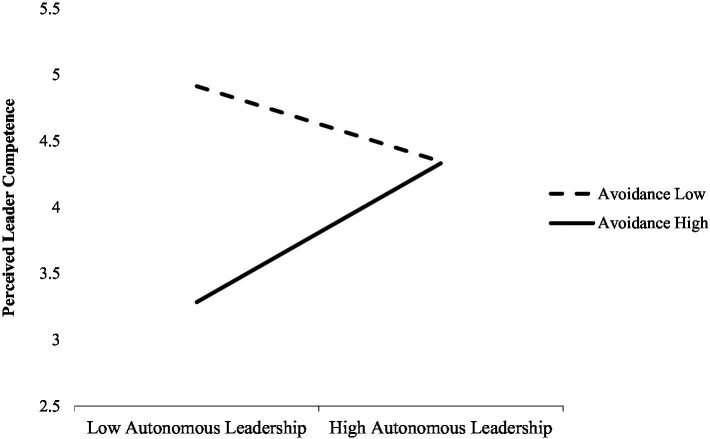
Regression of the interaction of avoidance attachment and autonomous leadership on perceived leader competence conditions (Study 2).

Avoidant attachment significantly and negatively related to the perception of leader competence (*b* = −0.27, *p* < 0.001) as a main effect. Moreover, in the high autonomous leader condition, avoidant attachment was related significantly and positively to perceived leader competence. To gain a better understanding of this interaction, we conducted a simple slopes test (see Figure 2), using the full range of the moderator to plot our results (Bauer and Curran, 2005).

In line with H2, regarding high avoidant attachment, there was a significant increase in the ratings of perceived leader competence between (high/low) autonomous leadership conditions (simple slope = 1.049, *t* = 2.074, *p* = 0.039). In low avoidant attachment, the difference between (high/low) autonomous leadership conditions was not significant (simple slope = −0.57, *t* = −1.8, *p* = −0.073).

On the other hand, anxious attachment significantly predicted overall leader competence perceptions (*b* = −0.37, *p* < 0.001). However, the interaction between anxious attachment and the autonomous leader conditions was not significant (*p* > 0.10). The interaction of anxious and avoidant attachment showed a significant effect with regard to general attributions of leader competence (*b* = 0.08, *p* < 0.001) in the less autonomous leader condition.

## Discussion

Attachment theory in organizational settings has witnessed an intense research interest in recent years (Harms, 2011; Yip et al., 2018). Yet, a focus on the socio-cognitive processes that drive leader-follower interaction has been scarce. The present studies examined how followers’ avoidant and anxious attachment orientations can shape perceptions of autonomy in leader-follower interaction. Autonomy is a key characteristic of attachment dynamics that varies as a function of attachment orientations (Allen et al., 2002; McElhaney et al., 2009) and also a key personality characteristic of leadership perceptions across cultures (House et al., 2004). We reasoned that since attachment orientations involve working models (self-other perception schemas) based on interactions with autonomous or less autonomous leaders/caregivers, attachment orientations would guide individuals’ preference for a leadership style and related behaviors higher or lower in autonomy. Hence, the relationship between attachment and preference for specific leaders and leadership behavior would be a function of individuals’ sense of security or insecurity (Gillath and Hart, 2010).

Study 1 found that individuals with an anxious attachment orientation tend to hold a lower preference for an autonomous leadership style. Individuals who score higher on anxious attachment likely exhibit a higher preference for leaders who display positive approach-related behavior, that is, a leader who is less likely to be autonomous and independent and likely to be more consultative, collaborative, and team-oriented. By perceiving others as more capable, anxious individuals effectively may fulfill their own attachment needs and therefore increase proximity and dependence on others.

On the other hand, avoidant participants responded better to an autonomous leadership style. For example, using a vignette-based experimental design, Study 2 allowed to establish causal links between the activation of leadership schemas high or lower in autonomy and leader evaluations for persons differing in attachment orientations. Indeed, individuals who scored high on avoidant attachment had higher positive attributions and a higher preference for an autonomous leadership style (H1). In the presented case, these individuals attributed higher perceived leader competence to the highly autonomous leader description, and lower perceived competence to the less autonomous leader description. Confrontation with a description of a less autonomous leader seems to have activated avoidant individuals’ affiliation-related thoughts and behavioral strategies, and have led them to judge this fictional leader as much less competent than the highly autonomous leader who better fitted their own ideal leader expectations. This lends support to the importance of attachment orientations and related working models in leader perception (Keller, 1999; Hansbrough, 2012). Avoidant individuals are self-reliant and autonomous and have generally negative views of others, based on previous consistent socialization experiences that distance one-selves from other attachment figures (Collins and Feeney, 2004). Hence, avoidant persons seem to have developed implicit leadership schemas that tend to favor self-reliant, autonomous, and self-governing leaders. Such leaders would provide a closer match and a better leader-follower fit to avoidant individuals’ ideal leader prototype.

In Study 2, we also found a significant interaction effect between anxious and avoidant attachment. Such an interaction effect is usually considered equivalent to higher fearful attachment orientation, an attachment orientation also characterized by a higher degree of anxious attachment (Brennan et al., 1998). Since fearful attachment involves a higher level of anxiety, this could be taken as evidence partly supporting our expectations regarding anxious followers’ ILTs.

These key findings add to the discussion of leader identity and how leader identity is shaped as a result of leader-follower fit (DeRue and Ashford, 2010; Junker and van Dick, 2014). Work on leader identity has identified followers’ ILTs (DeRue and Ashford, 2010) critically influence the granting (and claiming) of leader identity to others who match followers’ ILTs. Self-identity marks another important antecedent in the evaluation of leader-follower relationships. Specifically, Jackson and Johnson (2012) attest that follower self-identity “may moderate the effects of […] leadership” (p. 488) so that some followers will respond more favorably to certain leadership styles than others, depending on relational leader-follower fit. Hence, attachment orientations could provide additional, individual-level insights in the formation and granting of leader identity under the assumption of leader-follower fit. By examining how individual differences in attachment orientations can indirectly influence the ideal leader categorization process, the present set of studies contribute to the literature on leader-follower fit and respond to calls for more research on this topic (Shondrick et al., 2010; Epitropaki et al., 2013).

Finally, the variation of results between studies (a trend in support of H1 in Study 1 and H2 in Study 2), could be due to either the non-static character of leadership cognitions overall (Lord et al., 2001) or organizational or contextual factors that may interact with participants’ attachment orientations in determining their implicit leadership schemas. Future research could examine contextual processes that make shifting to different modes of attachment-related implicit leadership schemas possible.

The results have some key practical applications and theoretical implications. Leader-follower fit is a key antecedent to work performance (Martin et al., 2016). Given the increasing multicultural character of work teams and autonomy being a key facet of leader schemas cross-culturally (House et al., 2004), examining leader-follower fit in terms of attachment related ILTs can increase accuracy in predicting group cohesiveness and effectiveness in international working teams. On the theory level, there is an increasing awareness on the interplay between insecure attachment and cultural orientations especially around the independent/interdependent cultural dimension (Kafetsios and Gruda, 2018; Kafetsios, 2021). This evidence underline the need to extend our understanding of how followers’ attachment orientations interact with leader autonomy traits (e.g., independent self-construal) with respect to relational and team outcomes at work. Therefore further cross-cultural research on this topc is distinctly needed.

## Limitations and Future Research

This study is not without limitations. First, in both studies we relied on an online participant pool provided by Amazon MTurk. However, previous studies have shown that the MTurk population is similar to traditional participant pools (Paolacci and Chandler, 2014). Likewise, although the degree of control in an online environment is lower than traditional experiments conducted in the laboratory, the inclusion of various stated attention checks and robustness checks as described in the paper, provides us with confidence that the results are likely similar to expected results form a traditional participant pool completing surveys and the respective experiment in a laboratory setting.

Second, in Study 1 we found an association between attachment and the latent factor “Autonomy.” It is important to keep in mind that this factor originally was considered in the GLOBE study, a cross-cultural study of leadership. Hence, we acknowledge that the observed effects might present differently across cultures due to the association between attachment schemas and national cultures. As mentioned above, we encourage future research to examine in greater detail potential cross-cultural differences in the preference for autonomous leadership styles.

## Conclusion

This study addresses a previously understudied link between followers’ attachment orientations and autonomy-related ILTs. Using a diverse set of methods, our findings suggest that followers’ attachment schemas can shape the leader influence process depending on followers’ attachment-related leader perceptions and the degree of autonomy exhibited by the respective leader.

## Data Availability Statement

The raw data supporting the conclusions of this article will be made available by the authors, without undue reservation.

## Ethics Statement

The studies involving human participants were reviewed and approved by EMLYON Business School. The patients/participants provided their written informed consent to participate in this study.

## Author Contributions

DG: conceptualization, methodology, formal analysis, writing-original draft preparation. KK: investigation, writing-reviewing, and editing. All authors contributed to the article and approved the submitted version.

## Conflict of Interest

The authors declare that the research was conducted in the absence of any commercial or financial relationships that could be construed as a potential conflict of interest.

## Publisher’s Note

All claims expressed in this article are solely those of the authors and do not necessarily represent those of their affiliated organizations, or those of the publisher, the editors and the reviewers. Any product that may be evaluated in this article, or claim that may be made by its manufacturer, is not guaranteed or endorsed by the publisher.

## Footnotes

^1^Throughout this paper, we are solely concerned with ideal (or preferred) leader prototypes, not typical leader prototypes.
